# Using a 3-step semi-automated image analysis pipeline to quantify differences in VE-cadherin junction morphology in lymphatic endothelial cells in vitro

**DOI:** 10.64898/2026.10.05.756815

**Published:** 2026-10-06

**Authors:** Stephen Larson, Annabelle Feller, Catherine Whittington

## Abstract

Lymphatic endothelial cell (LEC) function is strongly tied to transitions between button- and zipper-like junctions that control fluid flow into vessels. Published methods quantifying junction morphology are limited to single cell analysis or use few quantitative metrics to describe junctions across LEC monolayers. Therefore, a novel 3-step image analysis pipeline was developed to identify new metrics that quantify differences in button- and zipper-like junctions in LECs. The pipeline used Cellpose, CellProfiler, and Fiji to analyze VE-cadherin expression across lymphatic vessels within a 3D microfluidic chip and quantify VE-cadherin radial intensity, number of objects, object size, branchpoints, endpoints, and junction thickness. Tumor necrosis factor (TNF)-α exposure produced button-like junctions, and unexposed cells were zippered. Radial intensity was heightened at the cell membrane in zipper-like junctions while perinuclear intensity increased for button-like junctions. Increased branchpoints, endpoints, objects per cell, and junction thickness were associated with junction buttoning, while increased object size was associated with junction zippering. Principle component analysis results reinforced these trends and identified metric combinations that represented each junctional state. Overall, this semi-automated pipeline quantified differences in VE-cadherin junction morphology across LEC monolayers and generated new quantitative metrics to expand the tools available for studying junction morphology in lymphatic vasculature.

## Full Text Availability

The license terms selected by the author(s) for this preprint version do not permit archiving in PMC. The full text is available from the preprint server.

